# Letter to the editor: Highly pathogenic influenza A(H5N1) viruses in farmed mink outbreak contain a disrupted second sialic acid binding site in neuraminidase, similar to human influenza A viruses

**DOI:** 10.2807/1560-7917.ES.2023.28.7.2300085

**Published:** 2023-02-16

**Authors:** Erik de Vries, Cornelis AM de Haan

**Affiliations:** 1 Section Virology, Department Biomolecular Health Sciences, Faculty of Veterinary Medicine, Utrecht University, Utrecht, the Netherlands

**Keywords:** influenza A virus, H5N1, mink, neuraminidase, second sialic acid binding site, avian influenza, highly pathogenic avian influenza virus

**To the editor**: We read with much interest the article by Agüero et al. [1], reporting a highly pathogenic influenza A(H5N1) virus outbreak in farmed minks in northwest Spain in October 2022. Genetic characterisation of the viruses showed that they resembled the A/gull/France/22P015977/2022-like genotype within clade 2.3.4.4b. Considering efficient airborne transmission of human viruses among ferrets (reviewed in [2]), the concern is raised that adaptation of avian viruses to mink may provide a first step towards potential human-to-human transmission.

The authors focused on the amino acid substitution T271A in polymerase subunit PB2, which is known to enhance polymerase activity of influenza A virus (IAV) in mammalian host cells similar to the seminal PB2 E627K mutation. They also show that the only substitution in haemagglutinin (HA) in comparison with closely related avian strains is I390M. This mutation is in the stem region of HA and might be associated with altered HA stability and transmissibility but as of yet has no known biological relevance. Mutations previously associated with increased human-type alpha 2,6-linked sialic acid receptor binding and/or airborne transmission in ferrets [2] were still limited to the lack of a glycosylation site at position 158. Substitutions F74S and V163L in neuraminidase (NA), which are located at sites of unknown functional importance, also distinguished the viruses in minks from closely related avian viruses. Remarkably, all four viruses from minks contained a methionine at position 396 located in the second sialic acid binding site (2SBS) in NA (for a review on the 2SBS, see [3]). Influenza A(H5N1) viruses predominantly carry an isoleucine at this position, which is critical for efficient binding of sialic acid to the 2SBS [3,4]. 

Phylogenetic analysis using Nextstrain (https://nextstrain.org, accessed: 1 Feb 2023 [5]) indicated that the I396M substitution, seldom observed in other H5N1 viruses, was acquired in an avian host immediately before emergence of the H5N1 virus in mink. The analysis moreover showed that substitution S369I, which is at the position of a sialic acid contact residue in the 2SBS, was obtained immediately before the acquisition of I396M. Substitution of residues at positions 369 and 396 has been shown to negatively affect N1 activity [3,4], herein referred to as residues 372 and 400. A functional 2SBS promotes NA activity, presumably by bringing multivalent sialic acid substrates in closer proximity of the catalytic site. A functional 2SBS, which specifically binds to alpha 2,3-linked sialic acid receptors, has been lost in all human (pandemic) viruses and in several other viruses adapted to mammalian host species. Notably, whereas a 2SBS is conserved in nearly all avian IAVs [3], it is disrupted in some avian influenza A(H7N9) and A(H9N2) isolates. This preceded the acquisition of mutations in HA that decreased binding to avian-type alpha 2,3-linked sialic acid receptors. It is possible that this concerted evolution of NA and HA is driven by the need to preserve an optimal HA-NA balance. At the same time, such compensatory mutations may also increase binding to human-type receptors [3], as was recently demonstrated for H5N1 viruses [6].

Disruption of the 2SBS in H5N1 viruses in minks is thus far an unreported feature that they have in common with human-adapted influenza A viruses. Loss of the 2SBS may drive selection for changes in the receptor binding properties of HA, possibly resulting in increased binding to human-type receptors. Such changes in HA are expected to promote replication and transmission in mammalian hosts, including humans. Preservation or loss of the 2SBS is likely a viral host range determinant and could be an early adaptation signal that should be included in analysis of the pandemic potential of emerging IAVs.

## References

[1] Agüero M Monne I Sánchez A Zecchin B Fusaro A Ruano MJ Highly pathogenic avian influenza A(H5N1) virus infection in farmed minks, Spain, October 2022. Euro Surveill. 2023;28(3):2300001. 10.2807/1560-7917.ES.2023.28.3.2300001 36695488 PMC9853945

[2] Herfst S Imai M Kawaoka Y Fouchier RA . Avian influenza virus transmission to mammals. Curr Top Microbiol Immunol. 2014;385:137-55. 10.1007/82_2014_387 25048542

[3] Du W de Vries E van Kuppeveld FJM Matrosovich M de Haan CAM . Second sialic acid-binding site of influenza A virus neuraminidase: binding receptors for efficient release. FEBS J. 2021;288(19):5598-612. 10.1111/febs.15668 33314755 PMC8518505

[4] Du W Dai M Li Z Boons GJ Peeters B van Kuppeveld FJM Substrate binding by the second sialic acid-binding site of influenza A virus N1 neuraminidase contributes to enzymatic activity. J Virol. 2018;92(20):e01243-18. 10.1128/JVI.01243-18 30089692 PMC6158415

[5] Hadfield J Megill C Bell SM Huddleston J Potter B Callender C Nextstrain: real-time tracking of pathogen evolution. Bioinformatics. 2018;34(23):4121-3. 10.1093/bioinformatics/bty407 29790939 PMC6247931

[6] Du W Wolfert MA Peeters B van Kuppeveld FJM Boons GJ de Vries E Mutation of the second sialic acid-binding site of influenza A virus neuraminidase drives compensatory mutations in hemagglutinin. PLoS Pathog. 2020;16(8):e1008816. 10.1371/journal.ppat.1008816 32853241 PMC7480853

